# Spina-Bifida, with Report of Cases

**Published:** 1902-03

**Authors:** J. R Garner

**Affiliations:** Atlanta, Ga.


### SPINA-BIFIDA, WITH REPORT OF CASES.*

BY J. R GARNER, M.D.,

ATLANTA, GA.

Though occurring only once in about twelve hundred (1200) births, perhaps the chief congenital deformity associated with the spine and due to developmental arrest is spina-bifida.

Knowing, as we do, that the deformity is usually due to an arrest of development, also that the spinal cord and the skin are both developed from the epiblast, many have been prone to attribute its etiology to a failure of segmentation in some portion of this embryonic layer, thereby preventing the normal development of structures lying between the spinal cord and the integument. Among the reported cases there are some in which this condition may have been the origin of the deformity; for in them the elements of the cord, meninges, nerves, vertebral arches and the integument have been found intimately fused together in the sac wall. These cases, however, are very few. Far more frequently no such fusion exists; the cord and other nervous structures are found attached to the inner surface of the sac wall, but are by no means

---

*Read before the Atlanta Society of Medicine, February 20th 1902.

fused with it, whereas the skin may bear no relation to the tumor save as a covering. Then there are other cases in which the fluid accumulation takes place either in the arachnoid cavity or in the sub-arachnoid space and posterior to the cord. In such cases the cord is not involved in the tumor at all, and the developmental arrest more probably occurred at some later period than that of the epiblastic segmentation.

Usually the laminæ of one or more vertebræ are deficient, but this condition is not always to be found, for rarely the tumor protrudes as a hernia through the intervertebral foramen or notch. Still more rarely are reported cases in which the body of the vertebra was involved and the tumor presented anteriorly into the abdominal cavity.

According to the extent of nervous involvement, our cases are conveniently divided into three anatomical varieties: Syringomyelocele, meningmyelocele, and meningocele.

Syringomyelocele, the rarest and at the same time the form offering the least favorable prognosis, is usually located in the dorsal or the dorso-lumbar regions. Here the fluid accumulation is found within the central canal of the cord, and the sac wall contains not only the meninges but also the cord itself, nerves and other adjacent embryonic elements. The amount and nature of the cord involvement renders this form the one with which the associated paralyses are most frequently found, as well as hare-lip, hydrocephalus, talipes, and other congenital anomilies.

Where meningomyelocle exists the location is most frequently the sacro-lumbar region. In this variety, which is perhaps the one most frequently seen, the cord is found within the tumor, and, although attached to, is never fused into the sac wall.

Meningocele is at once the simplest and most favorable form in which the abnormality may manifest itself. Here the spinal membranes alone participate in the sac formation, the accumulation of fluid having taken place posterior to the cord, as above decribed.

The usual location of the various forms of spina-bifida has been given above; any position along the spine, however, may be assumed by either variety.

Dependent not only upon the class, but also upon the location and size of the tumor, are the associated paralyses. Though more frequently found in conjunction with syringomyelocele, they are at times exhibited with the other forms as well. When the sacral or the sacro-lumbar region is the seat of the deformity the corda-equina is the only portion of the cord likely to be involved, and this may be only partially so, rendering the extremity paralysis incomplete, and often permitting the bladder and the rectum to escape.

The natural tendency of the tumor is to increase in size so long as unobstructed by surgical means. This increase may be more or less rapid, and, under favorable conditions, as when completely covered with healthy integument, they may assume almost any size. Among the largest so far reported was one twenty-two (22) inches in circumference. Not always, though, is the skin covering complete, or even if so it may be very thin or even ulcerated, and the patient is, in either case, in constant danger of rupture resulting in death from convulsions due to the rapid draining away of the cerebro-spinal fluid or to infection.

Though death usually comes quite early in life, there are a few cases in which the patient has reached adult life without treatment, experiencing no greater inconvenience than that caused by the size and position assumed by the tumor.

The treatment of spina-bifida ranges from mere temporising measures, such as simple bandage pressure, to excision of the sac. Though there are some who still support and practice the operation of withdrawing fluid from the tumor, the injection of Morton's fluid, and other similar measures, the larger number of surgeons prefer excission as an operation giving altogether more satisfactory results, while entailing no greater danger of shock or infection than the tapping process. A few cases of spontaneous recovery have been reported.

Three cases of spina-bifida have come under my observation:

The first patient was a male child delivered by myself The tumor, a meningocele, about half as large as the child's head, was in the cervical region and extended upward underneath the lower portion of the scalp. The size and position of the tumor greatly retarded labor. The mother was a quadripara, all previous labors having been easy. Talipes of the left foot was the only associated deformity, otherwise the child was in good condition, manifesting no nervous complications.

On the ninth day the tumor had grown somewhat larger and become quite tense, so that it became necessary to remove a portion of the fluid. A small trocar was inserted into the base of the tumor and near the mid-line and about three (3) drachms of fluid allowed to escape. Morton's Fluid was not injected, owing to the proximity to the brain and fear of undue irritation. Once every six to eight days it became necessary to repeat the operation, the tumor having refilled and again become tense, until the thirty-ninth (39th) day, when meningeal symptoms developed, the child dying three days later. Subsequent examination showed the laminæ of the the third and fourth cervical vertebræ to be deficient.

The second case was a pedunculated meningocele about the size of a small orange and situated in the lower portion of the lumbar region. This patient was a well-developed female child about eight months old, there being no other deformity. The skin was dissected down to the base of the tumor, the sac tied off (as there was no nervous involvement), the stump returned to the spinal canal, and the wound closed with interrupted silk sutures. Only one vertebra was involved, but no effort to repair this was made other than to freshen the ununited ends of the laminæ. The patient made a good recovery, and when seen five months later was in the best physical condition, showing no signs of return or of nervous irritation.

A meningomyelocele in a child three months old was the last. The tumor, located in the lumbo-sacral region, measured about two and a half inches in diameter, and was completely covered by integument; about three fourths of an inch to the left of the mid-line was a small cicatrix-like depression, and about the same distance to the right was a pedunculated growth about an inch in length and half an inch in diameter. When first seen the child seemed in good condition and exhibited no signs of deformity or paralysis; about a month later, however, nervous symptoms began to appear, the patient was very restless, sleeping but little and fretting almost continuously; nourishment was refused except in small quantities and at long intervals. The size of the tumor had increased but little, if any; pressure upon the fontane produced no change in its size, though crying caused it to become tense. Operation, by excision, was decided upon. An eliptical incision was made so as to include both the cicatrix-like depression and the protuberance in the flap; this was next dissected from the tumor and the tumor itself cleared to its base; to prevent escape of the cerebro-spinal fluid while the sac was opened, the neck was grasped with forceps. The cord and such nerves as were involved in the tumor were carefully returned to the spinal canal, the sac dissected away and the edges of the meninges sutured together. Three laminæ (those of the first sacral and last two lumbar vertebræ) were deficient, but no attempt was made at repairing them. An interesting feature, noted by the anesthetist, was that during the dissection whenever pressure was made upon the tumor the pupils would dilate widely. For a while the patient seemed in good condition, but on the third night convulsions set in and death followed in a few hours.

Although the mortality in operative cases of spina-bifida is large, most authorities approve of the radical operation. While it is true that a few cases may have reached adult life without operative interference, this is the exception, rupture or infection usually occurring in the early months of life and result in death. Those few exceptional cases who live longer and are refused operative relief usually suffer from the associated paralyses which in some instances do not manifest themselves for some time after birth.

